# A Study of Prevalence of Metabolic Syndrome in Premenopausal Women With Female Pattern Hair Loss: A Case-Control Study

**DOI:** 10.7759/cureus.68039

**Published:** 2024-08-28

**Authors:** Aravind Reddy, Kirti S Deo, Niranjana S Pillai, Priyanka Patil, Pooja Chaurasia, Namratha Puttur, Kshitiz Lakhey

**Affiliations:** 1 Dermatology, Dr. D. Y. Patil Medical College, Hospital and Research Centre, Pune, IND

**Keywords:** hair follicle, bmi, waist circumference, metabolic syndrome, fphl

## Abstract

Background and objective

Female pattern hair loss (FPHL), also known as androgenetic alopecia (AGA), is a condition where the hair follicles of genetically susceptible women gradually shrink and become thinner, leading to hair loss in a particular pattern. Metabolic syndrome (MS) is a collection of conditions that co-occur, increasing the risk of heart disease, stroke, and type 2 diabetes. This study aims to determine the prevalence of MS in premenopausal women in patients with FPHL.

Methods and materials

We conducted a case-control, hospital-based observational study at our institution for a period of two years, which included 62 patients, with 31 cases (patients with FPHL) and 31 controls (patients without FPHL).

Results

In some cases, the mean age was 29.81 years, while in controls, it was 28.84 years. The mean waist circumference (WC) in cases was 81.9 +/- 11.75 cm, and in controls, it was 72.65 +/- 8.86 cm, with a statistically significant p-value of 0.001. In some cases, the mean body mass index (BMI) was 26.28 +/- 4.09 kg/m^2^, and in controls, it was 24.52 +/- 2.78 kg/m^2^, with a statistically significant p-value of 0.013. Between cases and controls, there was no significant difference in the homeostatic model assessment of insulin resistance (HOMA-IR), fasting blood glucose (FBG), fasting triglyceride levels, fasting HDL, and fasting insulin levels.

Conclusion

The study found a significant association between WC and BMI in patients with FPHL in premenopausal women. This highlights the need for early screening and preventive measures for MS in women presenting with FPHL.

## Introduction

Female pattern hair loss (FPHL) also called androgenetic alopecia (AGA) is marked by a widespread decrease in density of hair across the crown and frontal scalp, while the frontal hairline remains intact. It has been linked to insulin resistance and other components of MS. These factors result in impaired blood flow which damages hair follicles and leads to hair loss [1,2].

FPHL can manifest in several different ways, including bitemporal thinning, frontal accentuation (Christmas tree pattern), frontotemporal recession (which is rather unusual), and diffuse central thinning [3].

MS refers to a group of conditions that involve cardiovascular and diabetes-related phenotypes [4]. The diagnosis of MS requires meeting three out of five criteria, as outlined in the ATP III clinical recommendations, which include a) waist circumference (WC) >102 cm (40 inches) for males and >88 cm (35 inches) for females; b) triglyceride levels: ≥150 mg/dL; c) HDL cholesterol: ≥40 mg/dL for males and <50 mg/dL for females; d) blood pressure: ≥130/≥85 mm Hg; and e) fasting glucose levels ≥110 mg/dL [5].

To assess whether components of MS significantly contribute to the development of FPHL, we conducted a case-control study comparing the prevalence of FPHL in premenopausal women with MS to those without the condition.

## Materials and methods

A case-control study was conducted over a period of two years at a tertiary care hospital in India. The study included patients attending the dermatology outpatient department, and the calculated sample size was 62, divided into two groups: 31 cases and 31 controls.

Ethics clearance was obtained from the institute’s ethics committee prior to the commencement of the study. The inclusion criteria included clinically diagnosed FPHL aged between 15 and 45 years. Age and sex were matched for controls without FPHL, excluding those with thyroid disorders and psoriasis. Exclusion criteria comprised individuals under 15 or over 45 years of age, pregnant or lactating women, patients who refused consent, and women who had attained menopause.

Data were collected using predesigned and pretested proforma. Socio-demographic and clinical information was obtained from both patients and controls. The purpose of the study was thoroughly explained to the participants and their relatives, and written informed consent was obtained, ensuring the confidentiality of all data. Blood samples were collected to analyze fasting blood glucose (FBG), fasting lipid profile (FLP), and fasting serum insulin levels.

For data analysis, information was entered into MS Excel (Microsoft Corporation, Redmond, Washington) and analyzed using SPSS version 27.0 (IBM SPSS Statistics for Windows, Armonk, NY). The student’s t-test was employed for quantitative data analysis, while the Chi-square test was used for comparing qualitative data. Quantitative data were presented as mean ± standard deviation (SD), and qualitative data were expressed as frequencies. Pearson's correlation test was applied, with a p-value of 0.05 considered statistically significant.

## Results

The study enrolled a total of 31 cases and 31 controls. In the case group, the mean age was 29.81 years, while in the control group, it was 28.84 years. Occupationally, the case group consisted of 16 homemakers, 10 students, and 5 engineers, whereas the control group had 13 students, 12 homemakers, and 6 engineers. The mean WC was statistically higher in cases (81.9 cm) compared to controls (72.65 cm), with a p-value of 0.001. Similarly, the mean body mass index (BMI) was statistically significantly higher in cases (26.28 kg/m^2^) than in controls (24.52 kg/m^2^) with a p-value of 0.013 (Table 1). However, regarding several other parameters, the difference between cases and controls was not statistically significant (p=0.995), mean fasting triglyceride level was 108.97 mg/dL in cases and 147.61 mg/dL in controls (p=0.995), mean fasting HDL was 43.97 mg/dL in cases and 38.65 mg/dL in controls (p=0.113), mean FBG was 95.26 mg/dL in cases and 88.52 mg/dL in controls (p=0.084), mean fasting insulin level was 9.92 mIU/L in cases and 6.94 mIU/L in controls (p=0.029), and mean homeostatic model assessment of insulin resistance (HOMA-IR) was 2.39 in cases and 1.61 in controls (p=0.073).

**Table 1:** Comparison of cases and controls in various studies with factors like age, WC, BMI, HOMA-IR, FBG, TG, HDL-C, and insulin The cases and controls of our study are being compared with three studies: Marwa et al., Rather et al., and El-Sayed et al. [6-8]. FBG: fasting blood glucose; TG: fasting triglycerides; BMI: body mass index; WC: waist circumference; HDL-C: high-density lipoprotein cholesterol; HOMA-IR: homeostatic model assessment of insulin resistance

Parameter	Study	Cases	Controls	Significant difference
Age (years)	Current study	29.81±7.7	28.87±8.62	
	Marwa et al. [6]	26.8±8.3	29.9±7.3	
	Rather et al. [7]	25.5	25.73	
	El-Sayed et al. [8]	27.8±7.9	26.8±7.3	
WC (cm)	Current study	81.9±11.75	72.58±8.86	P<0.05
	Marwa et al. [6]	93.5±9.7	85.9±9.9	P<0.01
	El-Sayed et al. [8]	95±14.2	85.5±10.7	P<0.05
BMI (kg/m²)	Current study	26.28±4.09	24.46±2.78	P<0.05
	Marwa et al. [6]	28.1±4.3	25.8±5.4	P>0.05
	Rather et al. [7]	No significant difference	No significant difference	No
Metabolic parameters				
HOMA-IR, FBG, TG, HDL-C, and insulin	Current study	No significant differences	No significant differences	No
	Marwa et al. [6]	Higher insulin, FBG, HOMA-IR, lower HDL-C in cases		P<0.05
	Rather et al. [7]	Higher TG and lower HDL		Yes
	El-Sayed et al. [8]	Higher TC and LDL in cases		P<0.05

## Discussion

FPHL is characterized by a shortening of the anagen phase and a reduction in the size of the dermal papilla. Miniaturization involves a gradual progression of large, pigmented terminal hair follicles into vellus-like hair follicles that are short, thin, and non-pigmented. This process is central to FPHL. Due to the shortened anagen phase, the vellus-like hair follicles experience a reduced hair cycle, resulting in shorter and thinner hair shafts. Thus, the early conclusion of the anagen phase is a key factor in the progression of FPHL [1]. The Kenogen phase is a dormant period following the telogen phase and before the onset of the new anagen phase, during which the hair follicle remains inactive. The size of the dermal papilla influences the length and width of the hair follicle. Shrinkage occurs when the volume of the papilla diminishes, usually during the transition from the catagen phase to the formation of new hair [2]. While the exact cause of miniaturization is unknown, one possible mechanism is a reduction in papilla cells due to apoptosis. Widespread apoptosis of keratinocytes within the follicles leads to thinning of hair follicles during the catagen phase [1]. Apoptosis is triggered by an imbalance in factors including fibroblast growth factor 5, interleukin 1 alpha, PG-D2, transforming growth factor beta 1, and tumor necrosis factor alpha 1 and Wnt signaling pathway [1].

Lymphocyte infiltration has been identified as a contributing factor in the progression of both male pattern hair loss (MPHL) and FPHL [1,2]. An immunohistochemical analysis of samples from three males and one female with advancing baldness showed the presence of CD4+ T-cells at the hair follicle bulge, whereas control samples had minimal T-cell presence. Inflammatory T-cells at the follicular bulge likely disrupt the hair cycle and cause hair loss in MPHL and FPHL. A recent study examining scalp samples from 52 individuals confirmed lymphocytic folliculitis, a histological feature of both MPHL and FPHL [9]. The blood vessels around the proximal bulb provide nutrients to the hair follicle matrix cells, which are actively dividing and differentiating during the anagen phase, and help the hair follicle grow in size. The upper venule annulus is a vascular structure that permanently encircles the bulge region of the hair follicle. Researchers suggest that when perifollicular vascularization decreases, it cuts off the hair follicle’s nutrient supply, impeding the growth of hair matrix cells. Reduced blood flow to the hair follicle has been linked to follicle shrinkage in conditions like AGA [10]. The decrease in perifollicular vascularization and chronic inflammation together contribute to the pathophysiology of FPHL and MPHL. Hyperglycemia, which is associated with insulin resistance and other MS markers such as increased WC and BMI, may cause vascular damage contributing to FPHL [1].

In our study, we wanted to examine the role of various parameters of MS in the causation of FPHL. We compared multiple characteristics between cases and controls. The mean age of cases was 29.81±7.7 years, while that of controls was 28.87±8.62 years, which is similar to the studies conducted by Marwa et al., Rather et al., and El-Sayed et al. [6-8]. Our sample consisted primarily of homemakers in the case group and students in the control group.

The mean WC was significantly higher in cases (81.9±11.75 cm) than in controls (72.58±8.86 cm), with similar findings seen by El-Sayed et al. finding central obesity (WC ≥88 cm) in 75.5% of cases vs. 35.5% of controls, with mean WCs of 95±14.2 cm and 85.5±10.7 cm, respectively (P<0.05), and Marwa et al. reported a significantly higher WC in cases (93.5±9.7 cm) compared to controls (85.9±9.9 cm; P<0.01) [6,8]. Both men and women who had a higher WC showed a 50% higher chance of mortality. Visceral fat significantly correlates with WC, making it a potential risk factor for visceral fat-related mortality [11]. Larger adipocytes usually have higher levels of pro-inflammatory factors like leptin, IL-6, IL-8, and monocyte-chemotactic protein-1 (MCP-1). Additionally, they have lower levels of adiponectin and IL-10, which are adipokines that aid the body in responding to insulin and breaking down fat more quickly in response to catecholamine stimulation [12]. Higher WC remains associated with a greater risk of death, even after accounting for BMI. This suggests that mortality, irrespective of overall body weight, is associated with visceral fat [11].

In our study, we found the BMI of cases was higher (26.28±4.09 kg/m²) than that of controls (24.46±2.78 kg/m²) with a significant difference. However, Marwa et al. and Rather et al. found no significant difference in BMI between cases and controls [6,7]. A higher BMI is consistently associated with an elevated risk of type 2 diabetes [13]. Obesity is often related to MS, a combination of high blood sugar, elevated lipid levels, and high blood pressure, which significantly contributes to the development of type 2 diabetes [14-16].

No significant differences were found in HOMA-IR, FBG, fasting triglycerides (TG), fasting high-density lipoprotein cholesterol (HDL-C), or fasting insulin levels between cases and controls, similar to the findings of El-Sayed et al., but they found higher total cholesterol (TC) and LDL levels in cases (P<0.05) compared to controls [8]. However, Rather et al. reported significant differences in hypertriglyceridemia and lower HDL levels among cases than controls [7]. Marwa et al. reported significantly higher fasting insulin, FBG, and HOMA-IR, as well as lower HDL-C in cases compared to controls (P<0.05) [6].

The study limitations involved a small sample size, which weakened the statistical power and affected the robustness of the findings, the use of only the Savin scale for case classification, the exclusion of disorders such as polycystic ovarian syndrome and thyroid dysfunction, and the lack of necessary in-depth evaluation of hormonal assessment.

## Conclusions

Our study shows that premenopausal women who have FPHL are prone to have MS. The findings of our study suggest a possible association between FPHL and MS components, as women with FPHL had higher WC and BMI than controls. There were no statistically significant differences observed for any measures, including fasting insulin levels, lipid profiles, and blood glucose levels. Further studies with greater sample sizes and comprehensive hormonal measures are needed to elucidate the connection between FPHL and MS.
